# Comparative genomic analysis of the flagellin glycosylation island of the Gram-positive thermophile *Geobacillus*

**DOI:** 10.1186/s12864-016-3273-2

**Published:** 2016-11-14

**Authors:** Pieter De Maayer, Don A. Cowan

**Affiliations:** 1 School of Molecular and Cell Biology, University of the Witwatersrand, Private Bag 3, Wits, 2050, Johannesburg, South Africa; 2 Centre for Microbial Ecology and Genomics, Genomics Research Institute, University of Pretoria, Pretoria, 0002 South Africa

**Keywords:** *Geobacillus*, Flagellin, Post-translational modification, Glycosylation, Glycosyltransferase, Pseudaminic acid

## Abstract

**Background:**

Protein glycosylation involves the post-translational attachment of sugar chains to target proteins and has been observed in all three domains of life. Post-translational glycosylation of flagellin, the main structural protein of the flagellum, is a common characteristic among many Gram-negative bacteria and Archaea. Several distinct functions have been ascribed to flagellin glycosylation, including stabilisation and maintenance of the flagellar filament, motility, surface recognition, adhesion, and virulence. However, little is known about this trait among Gram-positive bacteria.

**Results:**

Using comparative genomic approaches the flagellin glycosylation loci of multiple strains of the Gram-positive thermophilic genus *Geobacillus* were identified and characterized. Eighteen of thirty-six compared strains of the genus carry these loci, which show evidence of horizontal acquisition. The *Geobacillus* flagellin glycosylation islands (FGIs) can be clustered into five distinct types, which are predicted to encode highly variable glycans decorated with distinct and heavily modified sugars.

**Conclusions:**

Our comparative genomic analyses showed that, while not universal, flagellin glycosylation islands are relatively common among members of the genus *Geobacillus* and that the encoded flagellin glycans are highly variable. This suggests that flagellin glycosylation plays an important role in the lifestyles of members of this thermophilic genus.

**Electronic supplementary material:**

The online version of this article (doi:10.1186/s12864-016-3273-2) contains supplementary material, which is available to authorized users.

## Background

While long considered to be specific to eukaryotes, protein glycosylation is now known to be common in both Bacteria and Archaea, with even greater versatility in both glycan structure and composition observed in prokaryotic cells than in their eukaryotic counterparts [1]. This protein modification has a substantive effect on both the structure and function of the protein [2]. A large number of target proteins for posttranslational glycan modification have been identified, and include surface proteins such as pili, lipoproteins, adhesins and the surface layer proteins in many Archaea and Gram-positive bacteria, as well as secreted proteins such as antigens and pathogenicity effectors [1, 2]. Two discrete mechanisms for glycan transfer to the target protein have been identified, where the glycan chains are either assembled on a lipid carrier and transferred to the protein by oligosaccharyltransferases, or the sugars are sequentially attached by glycosyltransferases to the target protein [3]. Furthermore, glycans can be linked to distinct amino acids in prokaryotic proteins via *N*-linkage to the amide group of asparagines, or *O-*linked to the hydroxyl group of serine or threonine residues [3, 4].

The most extensively characterized post-translationally glycosylated protein is flagellin, the main structural unit of the flagellum, the whip-like appendage required for swimming motility. The C- and N-termini of flagellin proteins are very conserved, while the central region is highly variable and forms the surface-exposed portion of the protein [5]. Flagellin glycan linkages are generally restricted to this region and glycans are thus exposed to the environment [4, 6]. Flagellin glycosylation occurs in both Archaea and Bacteria, where in the former it occurs mainly in the *N*-linked conformation, while in the later the flagellin is generally *O*-glycosylated [7]. Diverse functions have been ascribed to flagellin glycosylation. In the Gram-negative bacterial pathogens *Campylobacter* and *Aeromonas*, the aquatic bacterium *Caulobacter crescentus* and the Gram-positive bacterium *Paenibacillus alvei*, flagellin glycosylation is imperative for assembly of the flagellum and flagellar motility [7–10]. By contrast, glycosylation gene deletion in the opportunistic human pathogen *Pseudomonas aeruginosa* and plant pathogen *Pseudomonas syringae* had no direct effect on assembly or motility [11, 12]. As flagellin is a highly immunogenic protein recognised by the host during infection, flagellin glycosylation in Gram-negative pathogens can facilitate immune evasion [13, 14]. Other purported functions of flagellin glycosylation include surface recognition, attachment and adhesion, biofilm formation, increased resistance against proteolytic degradation and virulence [15–17]. Similarly, in Archaea, glycosylation has also been shown to be essential for flagellar biosynthesis and motility in some species, while *N-*glycosylation of flagella has been suggested to contribute to their ability to survive under harsh environmental conditions [18].

While flagellin glycosylation is a well-documented feature in Gram-negative bacteria and Archaea, it has only been observed in a limited number of Gram-positive taxa, including members of the genera *Listeria, Clostridium, Butyrivibrio* and *Paenibacillus* [7, 8, 12]. Moreover, the molecular determinants of flagellin glycosylation have only been studied in one Gram-positive bacterium, *Clostridium botulinum* [19]. Members of the genus *Geobacillus* are Gram-positive, rod-shaped, aerobic, obligate thermophiles. This genus currently comprises 16 species which are commonly isolated from high temperature environments, including hot springs, oil wells and compost although they have also been isolated from more temperate environments. *Geobacillus* spp. have received extensive interest as the sources of a range of thermostable enzymes with various industrial and biotechnological applications [20–22]. Periodic acid Schiff (PAS) staining demonstrated that the flagellin of *Geobacillus stearothermophilus* NBRC12550^T^ is glycosylated [23]. Here, using comparative genomic analyses, we show the presence of flagellin glycosylation islands (FGIs) in the genome sequences of half of the 36 compared *Geobacillus* strains. These FGIs are highly variable, suggesting that these *Geobacillus* strains have the genetic potential to synthesise distinct, extensively decorated flagellin glycans. Finally, we discuss potential functional roles for flagellin glycosylation in *Geobacillus* spp.

## Results and discussion

### General properties of the *Geobacillus* FGIs

**Fig. 1:**
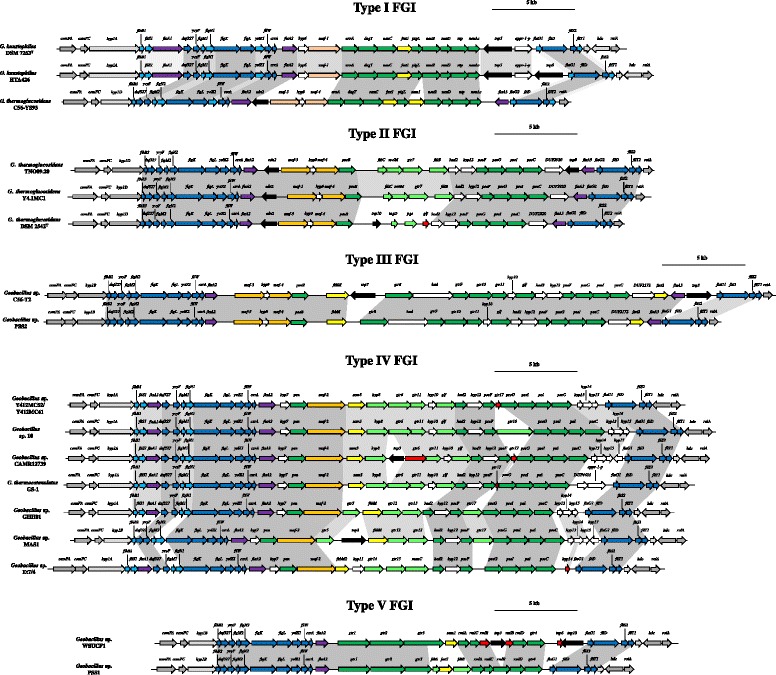
Schematic diagram of the flagellin glycosylation islands of FGI^+^ *Geobacillus* strains. The strains were clustered into five distinct types on the basis of the presence/absence of orthologs of the FGI proteins. The flanking genes are coloured in grey, while the flagellin genes and flagellum biosynthetic genes are indicated in *purple* and *blue*, respectively. Genes coloured in *green* are involved in glycosylation and glycan biogenesis, while *yellow arrows* denote those genes involved in further modification (formylation and methylation) of the flagellin protein and/or glycan chain and *orange arrows* represent the predicted *maf* genes. Genes coding for hypothetical proteins of unknown function, transposases and truncated genes are shown as *white*, *black* and *red arrows*, respectively. A scale bar indicates the predicted size of the FGIs

**Fig. 2:**
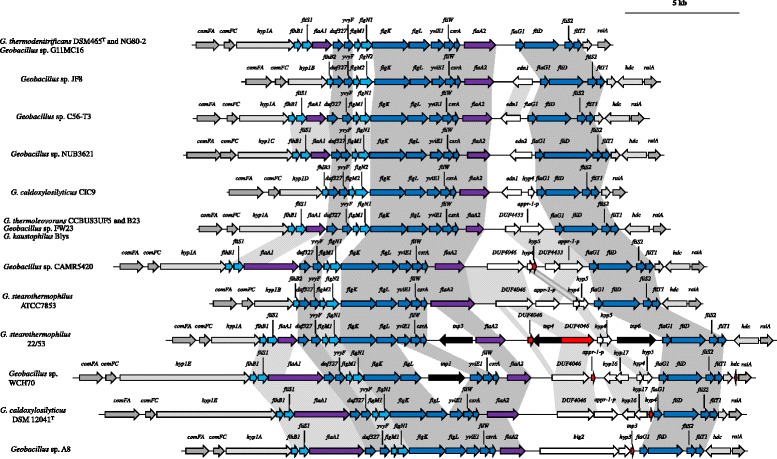
Schematic diagram of the flagellum biosynthetic locus in FGI^−^ *Geobacillus* strains. The flanking genes are coloured in grey, while the flagellin genes and flagellum biosynthetic genes are indicated in *purple* and *blue*, respectively. Genes coding for hypothetical proteins of unknown function, transposases and truncated genes are shown as *white*, *black* and *red arrows*, respectively. A scale bar indicates the predicted size of the regions

The complete and draft genomes of 36 *Geobacillus* strains were analyzed for the presence of genomic islands using the IslandViewer server [24]. A predicted genomic island was found to be integrated within a flagellar biosynthetic locus conserved in all sequenced *Geobacillus* strains. This locus is comprised of genes coding for the main flagellar filament subunit (*flaA1* and *flaA2*), filament cap protein (*fliD*) flagellar hook-filament proteins (*flgK* and *flgL*), the flagellar export proteins (*fliS, fliT, flhB*, *flgN*) and the anti-sigma factor (*flgM*) [25]. The genomic island is localized between the flagellin gene *flaA2* and *flaG*, which codes for a flagellar protein of unknown function. The island varies in size from 0.9 to 30.4 kb. The protein coding sequences (CDSs) for these regions were predicted, and in fifteen of the sequenced *Geobacillus* strains, CDSs coding for glycosyltransferases were present. Three additional strains did not encode glycosyltransferases in this locus, but did code for predicted motility-associated factors (Maf proteins). Orthologs of *maf* genes have been identified in *Aeromonas* spp., *Helicobacter* spp. and *Campylobacter* spp., with seven *maf* genes (*maf1-maf7*) occurring in the flagellin glycosylation locus of *Campylobacter jejuni* NCTC 11168 [26, 27]. The exact function of these Maf proteins remains unclear, although the genetic localisation of the *maf* genes, as well as the unglycosylated flagellin phenotypes of *maf* knock-out mutants, suggests a role in glycosylation [26, 27]. Molecular evidence for *Aeromonas caviae* suggests that the Maf proteins represent a novel family of flagellin glycosyltransferases [9]. The three *Geobacillus* strains lacking a glycosyltransferase gene, but with a *maf* gene in their flagellin loci, were thus considered to be FGI^+^ (Fig. 1). As such, 18 out of the 36 *Geobacillus* strains were considered to carry a flagellin glycosylation island (FGI^+^), while the remaining 18 strains were considered to be FGI negative (FGI^−^) (Fig. 2).

**Fig. 3:**
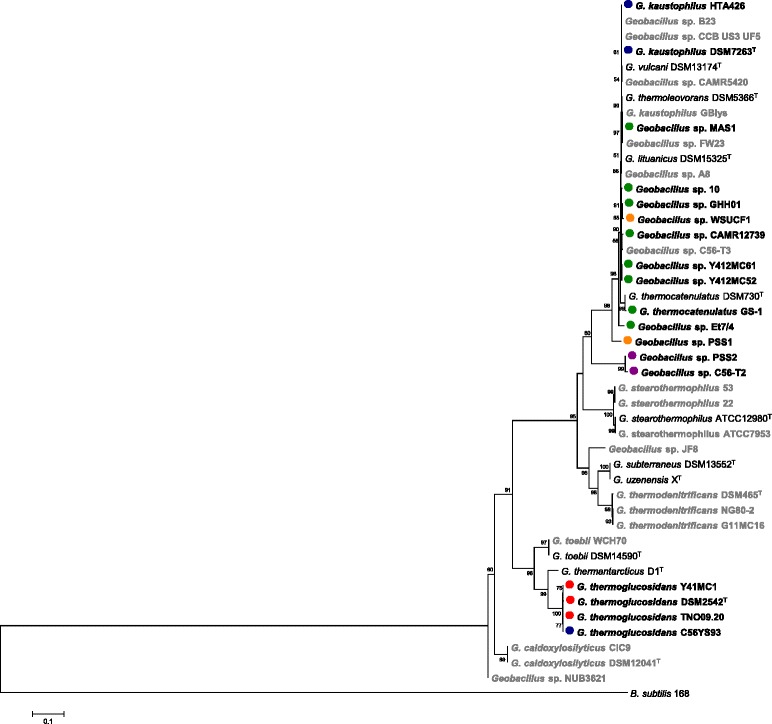
*recN* Maximum Likelihood phylogeny of the FGI^+^ and FGI^−^ *Geobacillus* strains. Those strains in which no FGI is present are shaded in grey. The FGI^+^ strains are indicated in bold with *blue* (I), *red* (II), *purple* (III), *green* (IV) and *brown* (V) dots indicating the respective group to which they belong. *B. subtilis* strain 168 was included as outgroup. Boot strap values (*n* = 1000 replicates) are indicated

A Maximum Likelihood phylogeny of the 36 *Geobacillus* strains, and type strains of each of validly described species, was constructed on the basis of the *recN* gene. This gene has been shown to result in similar branching patterns as 16S rRNA phylgeny, albeit with greater resolving power between closely related strains, and closely reflects the whole genome relatedness of *Geobacillus* species, as well as a range of other Gram-positive and Gram-negative taxa [28, 29]. This phylogeny (Fig. 3) shows the absence of a flagellin glycosylation island in some species for which more than one genome sequence is available, including *G. stearothermophilus* and *G. caldoxylosilyticus*, while FGIs are present in all four sequenced *G. thermoglucosidans* strains. By contrast, the *G. kaustophilus-thermoleovorans-vulcanii-lituanicus* clade showed a more random distribution in terms of the presence/absence of flagellin glycosylation islands (Fig. 3).

The *flaA2*-*flaG* intergenic region in the FGI^−^ strains is relatively small, ranging in size from 0.9 to 6.9 kb (average G + C content: 44.73%; 5.18% below genome average) and coding for between zero and six CDSs (Table 1). Among the genes within this region in the FGI^−^ strains *Geobacillus* sp. C56-T3, JF8 and *G. caldoxylosilyticus* CIC9 is a gene (*edn1*) coding for a LAGLIDADG family site-specific DNA endonuclease. A paralogous copy (*edn2*) is also found in this region in the FGI^−^ strain *Geobacillus* sp. NUB3621. This type of “homing” endonuclease catalyzes site-specific cleavage of DNA and subsequent repair by integration in the cleavage site [30]. Furthermore, a Poa1P-like macro domain (cd02901) protein (*appr-1-p*) is found in the FGI^−^ strains *Geobacillus* sp. CAM5420 and FW23, as well as *G. thermoleovorans* CCB-US3_UF5 and B23, and *G. kaustophilus* Blys. This domain plays a role in ADP-ribosylation of proteins which effect DNA excision repair [31]. The presence of these proteins within the genomic island of FGI^−^ strains suggests a potential mechanism of loss of FGI genes in the FGI^−^ strains. However, copies of the genes coding for the endonuclease are also present in the FGI^+^ strains *G. thermoglucosidans* TNO09.20 and Y4.1MC1 (*edn1*) and *G. thermoglucosidans* C56YS93 and DSM 2542^T^ (*edn2*), while orthologs of Appr-1-P are encoded in the FGIs of *G. kaustophilus* DSM 7263^T^ and HTA426 and *G. thermocatenulatus* GS-1.

**Table 1:** Flagellin glycosylation island metrics

Strain	Isolation source	Geographic location	Genbank Acc. # of containing contig	FGI Type	Genome average	Island G + C%	G + C% deviation	Size (kb)	# CDS
*G. thermodenitrificans* DSM465^T^	Sugar beet juice	Austria	AYKT01000009	-	49.05%	45.08%	−3.97%	0.9	0
*G. thermodenitrificans* G11MC16	Grass compost	USA	ABVH01000005	-	48.80%	45.77%	−3.03%	1	0
*G. thermodenitrificans* NG80-2	Formation water of oil well	China	CP000557	-	49.01%	46.30%	−2.71%	1.3	0
*Geobacillus* sp. C56-T3	Hot Spring	Nevada, USA	CP002050	-	52.49%	43.24%	−9.25%	1.6	1
*G. caldoxylosilyticus* CIC9	Hot Spring	Indonesia	AMRO01000028/052	-	44.17%	39.14%	−5.03%	2	2
*Geobacillus sp.* NUB3621	Soil	China	AOTZ01000009	-	44.38%	42.37%	−2.01%	2	1
*Geobacillus* sp. JF8	Bark compost	Okayama, Japan	CP006254	-	52.87%	46.52%	−6.35%	2.1	1
*Geobacillus* sp. FW23	Formation water of oil well	Gujrat, India	JGCJ01000045/075	-	52.24%	49.36%	−2.88%	3.1	2
*G. thermoleovorans* B23	Production water, subterranean oil reservoir	Niigata, Japan	BATY01000075	-	52.29%	49.36%	−2.93%	3.1	2
*G. kaustophilus Blys*	Hot Spring	Japan	BASG01000016	-	52.05%	49.35%	−2.70%	3.1	2
*G. thermoleovorans* CCB_US3_UF5	Hot Spring	Perak, Malaysia	CP003125	-	52.28%	49.36%	−2.92%	3.1	2
*Geobacillus* sp. CAMR5420	CAMR thermophile culture collection	University of Bath, UK	JHUS01000064	-	51.89%	39.70%	−12.19%	4.5	5
*Geobacillus* sp. A8	Deep mine water	Limpopo, South Africa	AUXP01000036	-	52.41%	46.24%	−6.17%	5.1	3
*G. stearothermophilus* ATCC7953	Underprocessed canned food	USA	JALS01000021/022	-	52.39%	41.28%	−11.11%	5.3	4
*G. toebii* WCH70	Compost	USA	CP001638	-	42.84%	40.76%	−2.08%	5.6	6
*G. caldoxylosilyticus* DSM 12041^T^	Soil	Australia	BAWO01000015/16/56	-	43.92%	40.33%	−3.59%	5.9	5
*G. stearothermophilus* 22	Hot Spring	Garga, Russian Federation	JQCS01000048/070/194	-	52.62%	45.46%	−7.16%	6.9	6
*G. stearothermophilus* 53	Hot Spring	Garga, Russian Federation	JPYV01000016/113/157	-	52.56%	45.46%	−7.10%	6.9	6
*G. kaustophilus* DSM 7263^T^	Pasteurized milk	USA	BBJV01000001/072	I	51.99%	36.60%	−15.39%	14.5	13
*G. thermoglucosidans* C56-YS93	Hot Spring	Obsidian, USA	CP002835	I	43.95%	34.60%	−9.35%	15.7	15
*G. kaustophilus* HTA426	Deep sea sediment	Mariana Trench	BA000043	I	52.09%	38.35%	−13.74%	16.5	14
*G. thermoglucosidans* TNO09.20	Dairy factory biofilm	Netherlands	CM001483	II	43.82%	35.00%	−8.82%	20.6	18
*G. thermoglucosidans* Y4.1MC1	Hot Spring	Yellowstone National Park, USA	CP002293	II	44.02%	34.83%	−9.19%	20.3	17
*G. thermoglucosidans* DSM 2542^T^	Soil	Kyoto, Japan	BAWP01000013	II	43.69%	36.16%	−7.53%	19.2	17
*Geobacillus* sp. PSS2	Dead, steaming tree	Kilauea Volcano, Hawaii	JQMN01000001	III	51.58%	36.93%	−14.65%	27	21
*Geobacillus* sp. C56-T2	Hot Spring	Nevada, USA	GC56T2_Contig257 ^a^	III	52.39%	38.95%	−13.44%	30.4	23
*Geobacillus* sp. Y412MC52	Hot Spring	Yellowstone National Park, USA	CP002442	IV	52.43%	44.58%	−7.85%	20.1	20
*Geobacillus* sp. Y412MC61	Hot Spring	Yellowstone National Park, USA	CP001794	IV	52.42%	44.58%	−7.84%	20.1	20
*G. thermocatenulatus* GS-1	Oil well	China	JFHZ01000063	IV	52.11%	45.20%	−6.91%	20.5	19
*Geobacillus* sp. CAMR12739	CAMR thermophile culture collection	University of Bath, UK	JHUR01000060	IV	52.21%	44.67%	−7.54%	21.2	22
*Geobacillus* sp. MAS1	Hot Spring	Pakistan	AYSF01000034	IV	52.21%	43.73%	−8.48%	21.5	20
*Geobacillus* sp. 10	Hot Spring	Yellowstone National Park, USA	CP008934	IV	52.71%	43.29%	−9.42%	22	20
*Geobacillus* sp. Et7-4	Geyser	El Tatio, Chile	JYBP01000003	IV	51.69%	41.96%	−9.73%	18.8	16
*Geobacillus* sp. GHH01	Botanical garden soil	Hamburg, Germany	CP004008	IV	52.28%	43.46%	−8.82%	18.9	18
*Geobacillus* sp. WSUCF1	Compost	Washington, USA	ATCO01000109/170/215	V	52.21%	39.44%	−12.77%	15.8	13
*Geobacillus* sp. PSS1	Dead, steaming tree	Kilauea Volcano, Hawaii	JPOI01000001	V	52.40%	38.13%	−14.27%	13.4	11

The sizes of the genomics islands for the FGI^−^ and FGI^+^ strains are indicated, as are the number of proteins (CDS) encoded in each and the difference in G + C content (%) from the genomic average. ^a^denotes the contig as per the Integrated Microbial Genome Database project (IMG ID 250801004) from which the data was obtained. The environmental source and geographical location from which each of the strains was isolated are indicated

The *flaA2*-*flaG* intergenic region in the FGI^+^ *Geobacillus* spp. is substantially larger than that of its FGI^−^ counterparts, ranging in size from 13.4 to 30.4 kilobases and coding for between 11 and 23 proteins. The G + C content (average = 40.33%) of this FGI is on average 10.32% lower than the mean genomic G + C content (Table 1), which indicates that this region was probably derived by horizontal acquisition.

### *Geobacillus* FGIs can be clustered into five distinct types which correlate poorly with the *recN* phylogeny

**Fig. 4:**
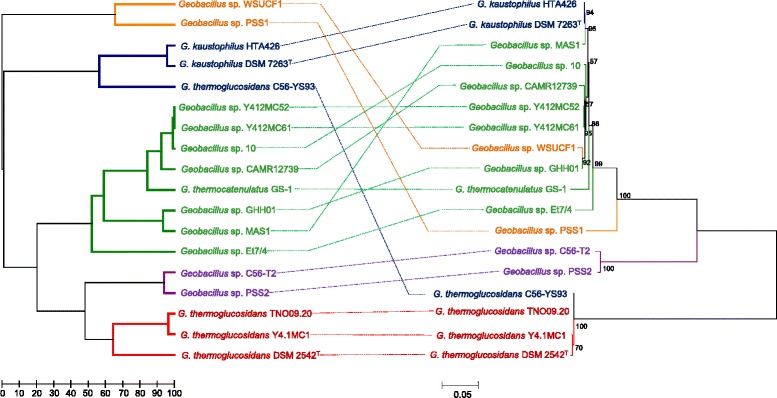
FGI typing dendrogram. A UPGMA dendrogram calculated on the basis of the presence/absence of FGI proteins is shown on the left, while a *recN* Maximum Likelihood phylogeny is shown on the right, with the branch and taxa colours reflecting the FGI types indicated in Fig. 3. Boot strap values (*n* = 1000 replicates) are indicated

Orthologs for each CDS encoded within the FGIs were identified using BlastP analyses of the CDS sets encoded on the island of the 18 FGI^+^ strains. On the basis of presence/absence of the 90 distinct CDSs encoded on the islands, a similarity matrix was constructed. This matrix was subsequently used to generate a UPGMA tree reflecting the similarity values between each of the compared FGI^+^ strains. Using a 50 % similarity cut-off value, the FGIs could be clustered into five distinct types, Types I-V (Fig. 1). The UPGMA tree was compared with a Maximum Likelihood phylogeny of the *recN* genes of the 18 FGI^+^ strains (Fig. 4). Only weak correlations between the FGI type and phylogeny could be observed. For example, while three of the sequenced *G. thermoglucosidans* strains encode Type I FGIs, *G. thermoglucosidans* C56-YS93 encodes a Type II FGI, while the Type V FGI-containing strains (*Geobacillus* sp. WSUCF1 and PSS1) are interspersed among the Type IV FGI strains in the *recN* phylogeny. This provides further evidence that the FGIs were derived through distinct horizontal acquisition events.

### *Geobacillus* spp. vary in both the number and type of flagellin genes

**Fig. 5:**
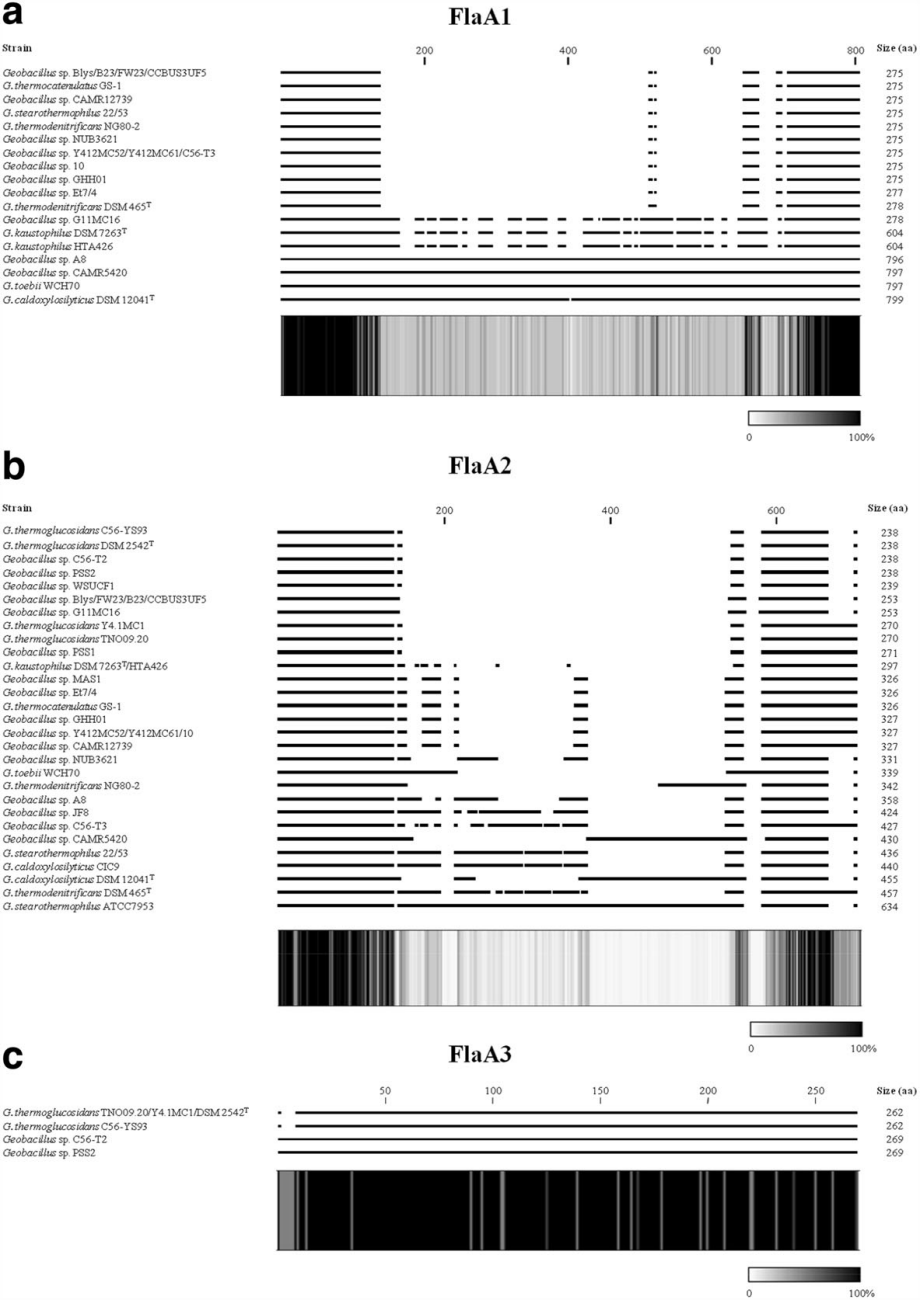
Alignments of the flagellin protein amino acid sequences FlaA1 (**a**), FlaA2 (**b**) and FlaA3 (**c**). The lengths of the flagellins are indicated on the right. The bar chart beneath each alignment shows the % conservation at each amino acid position, with *black* indicating the highly conserved residues, while *white* represents the non-conserved residues

Analysis of the *Geobacillus* FGI regions showed that they are flanked by up to three distinct flagellin (*flaA*) genes (Figs. 1 and 2), with 30 out of the 36 strains carrying two copies. One flagellin copy, *flaA2* is maintained in all *Geobacillus* strains, both FGI^−^ and FGI^+^, and is located at the 5' boundary of the FGIs. The FlaA2 protein is, however, highly variable, sharing only 49.01% average amino acid identity among the 36 compared strains, and ranging in length from 238 to 634 amino acids (Fig. 5a). FlaA1 is encoded on the genomes of 24 strains, including 15 FGI^−^ and nine FGI^+^ strains (two out of three type I FGI strains and seven out of eight type IV FGI strains), and is also highly variable, ranging in size from 275 to 799 amino acids, with an average amino acid identity of 61.92% among the 24 strains (Fig. 5b). The *flaA1* gene is flanked by a second copy of *fliS*, which codes for a flagellin-binding chaperone that facilitates flagellin export [32], which is also absent from those strains missing flaA1. The FlaA3 proteins are similar in size (262 to 269 amino acids) and are highly conserved at the sequence level (93.84% average amino acid identity). Alignment of the FlaA1 and FlaA2 amino acid sequences shows that extensive sequence conservation exists in both the N- and C-termini of these proteins (Fig. 5a and b), with a highly variable central region. A similar pattern has been observed in a range of both Gram-positive and Gram-negative bacteria, with the termini of the flagellin protein being membrane bound, whereas the central region represents the surface exposed region of the protein and is under positive selective pressure [5, 33]. No discernible pattern, in terms of protein length and sequence conservation of the FlaA1 and FlaA2 proteins, can be observed for the FGI^+^ and FGI^−^ strains, suggesting that the type of flagellin(s) produced is not a strict determinant of its post-translational glycosylation. By contrast, the third flagellin protein, FlaA3, is restricted to six FGI^+^ strains, including one Type I, three Type II and two Type III FGI strains (Fig. 5c). The presence of *flaA3* in strains with three different types of FGI, and the presence of this gene in only one of three strains with Type I FGI suggests, however, that this flagellin gene alone does not dictate its post-translational modification with a particular glycan.

The presence of two distinct flagellin genes in 30 of the analysed *Geobacillus* strains indicates that strains of this species may be capable of flagellin phase variation. This process has been observed in a number of Gram-negative pathogens including *Campylobacter jejuni*, *Salmonella enterica* and *Escherichia coli*, where two antigenically distinct flagellin genes are alternatively expressed [27, 34, 35]. As flagellin proteins represent potent antigens which can serve as a trigger for innate immune responses in both plant and animals, the phase-variable expression of a distinct flagellin can allow a pathogen to temporarily avoid cellular immunity [34–36]. Whether *Geobacillus* spp. are capable of phase variable expression of the distinct flagellin genes and the potential biological role of this trait in these environmental bacteria remains to be functionally determined.

### The *Geobacillus* FGIs carry genes for several distinct glycan biosynthetic pathways

Nineteen distinct glycosyltransferases (*gtr1-gtr18* and *manC*) are encoded within the flagellin glycosylation islands among the 18 FGI^+^ strains, with up to five distinct glycosyltransferases (Type III FGI strains *Geobacillus* sp. C56-T2 and PSS2) in the individual strains. The glycosyltransferases were classified into their respective Glycosyl Transferase families using the dbCAN Blast tool [37, 38]. Ten of the FGI glycosyltransferases belong to the GT2 family, eight to the GT4 family, while Gtr16 could not be classified in a particular family (GT0). Both the GT2 and GT4 family have transferase activities for a wide range of target sugars; and thus, the type of glycan transferred to the flagellin proteins can therefore not be inferred on the basis of glycosyltransferase type alone. One exception is the mannose-1-phosphate guanylyltransferase in the Type IV FGI strain *Geobacillus* sp. Et7/4, which shares 64.93% amino acid identity with the ManC enzyme in the S-layer glycosylation locus of *Aneurinibacillus thermoaerophilus* L420-91^T^ (AAS55729.1), suggesting the flagellin in *Geobacillus* Et7/4 is mannosylated. With the exception of the Type V FGI strains, the remaining 16 FGI^+^ strains encode four distinct Maf proteins. One Maf protein is found in two of the type I FGI strains, *G. kaustophilus* DSM 7263^T^ and HTA426 (*maf1*) and the eight type IV FGI strains (*maf4*). By contrast, the type I FGI strain *G. thermoglucosidans* C56-YS93 and all type II and III FGI strains encode two Maf proteins (*maf2* and *maf3*). If, as is predicted to be the case in *A. caviae* [9], these *maf* genes encode glycosyltransferases with unknown glycan substrates, this further confounds the roles of the distinct glycosyltransferases in *Geobacillus* flagellin glycosylation. However, a variety of enzymes for the biosynthesis of distinct sugars are encoded in the FGIs and, on this basis, some predictions on the putative sugar constituents of the *Geobacillus* flagellin glycans could be made.

The Type I FGIs encode orthologs of four proteins involved in the biosynthesis of *N-*acetyl neuraminic acid (NeuAc). NeuAc belongs to the nonulosonic acids, a diverse family of acidic nine-carbon backbone monosaccharides which also includes pseudaminic (Pse) and legionamic (Leg) acids [39]. NeuAc is incorporated in the polysialic capsule of *E. coli*, the lipo-oligosaccharide (LOS) of *Campylobacter jejuni* and LPS of *Leptospira* spp. [40, 41], but has not been observed as part of flagellin glycans. By contrast, Pse is frequently found as part of flagellin glycans, including in *Campylobacter* and *Helicobacter* spp. [7], and legionamic acid forms part of the glycans associated with the flagellins of *Campylobacter* spp. and *Clostridium botulinum* [19]. The UDP-GlcNAc 2-epimerase NeuC initiates the conversion of UDP-GlcNAc to ManNAc. Subsequently, NeuB condenses ManNAc and phosphoenolpyruvate before CMP-NeuNAc synthetase (NeuAc) activates the *N-*acetyl-neuraminic sugar [42, 43]. While the function of the fourth protein, the acetyltransferase NeuD, is unknown, it is predicted to play a role in the stabilisation of NeuB [42, 43]. The NeuAcBCD proteins encoded in the type I FGIs of *G. kaustophilus* DSM 7263^T^ and HTA426, and *G. thermoglucosidans* C56-YS93 share 47.98% average amino acid identity with their orthologs in the *E. coli* H708b O-antigen cluster (BAQ01507-1512) [44]. Interspersed among the Type I FGI *neuCBDA* genes are genes encoding an NAD-dependent epimerase (*arnA*), an aminotransferase (*degT*) and a nucleotidyltransferase (*ntp*). This has also been observed in *Leptospira interrogans* and these are predicted to be involved in the synthesis of neuraminic acid [45].

The Type II, III and IV FGIs (13 *Geobacillus* strains) contain genes coding for enzymes involved in the synthesis of 5,7-diacetamido-3,5,7,9-tetadeoxy-L-*glycero-α-*L*-manno*-nonulosonic acid (pseudaminic acid - Pse). The Pse biosynthetic pathway involves six enzymes. A bi-functional 4,6-dehydratase/5-epimerase (PseB) converts UDP-D-GlcNAc to UDP-4-keto-6-deoxy-L-AltNAc which is subsequently aminated at C4 by aminotransferase PseC and *N-*acetylated by the *N-*acetyltransferase PseH to form 2,4,6-tridoxy-2,4-NAc-L-altrose. UDP is cleaved from the sugar by UDP-sugar hydrolase PseG and it is pyruvylated by the pseudaminic acid synthase PseI. Finally, the cytidylyltransferase PseF adds cytidine monophosphate to produce the final CMP-Pse product [46–48]. With the exception of the *N-*acetyltransferase PseH, orthologs of four CMP-Pse biosynthetic enzymes (PseC, PseF, PseG and PseI) are encoded in the FGIs of all Type II, III and IV *Geobacillus* strains. These proteins share 52.69% average amino acid identity with PseC in the FGI of *Bacillus thuringiensis* subsp. *israelensis* ATCC35646 (RBTH_04255-4259), where they are likewise involved in biosynthesis of the pseudaminic acid precursor of the flagellin glycan [47]. In the Type II and III FGI strains, a gene coding for the bi-functional dehydratase/epimerase PseB (59.92% average amino acid identity to *C. jejuni* CJ1293) is present at the 5' end of the FGI. In *B. thuringiensis* ATCC356464, the initial conversion step of UDP-GlcNAc to UDP-4-keto-6-deoxy-L-AltNAc catalysed by PseB in *Campylobacter* and *Helicobacter* spp. is undertaken by two distinct enzymes, a UDP-GlcNAc 4-oxidase/5,6-dehydrogenase/4 reductase (Pen) and a UDP-6-deoxy-D-GlcNAc-5,6-ene 4-oxidase/5,6-reductase/-5-epimerase (Pal) [47]. Orthologs of Pen and Pal (RBTH_04253-4255: 66.0% average amino acid identity) are present in the FGIs of the Type IV FGI strains. The *pen* gene is localized at the 5' end of the Type IV FGI, while *pal* is located near the 3' end, in contrast to the pseudaminic acid biosynthetic locus in *B. thuringiensis* ATCC35646, where they are found adjacent to each other (Additional file 1: Figure S1). Alignment of the *Geobacillus* FGIs against the partial flagellin glycosylation locus of *C. jejuni* 81–176 (AY102662) also demonstrates extensive rearrangement of the pseudaminic acid biosynthetic genes within the *Geobacillus* FGIs (Additional file 1: Figure S1). A phylogeny constructed on the basis of the concatenated PseI and PseC protein sequences, reflects the distinct clustering of the *pseB*-containing (Type II and III FGI) and *pen* and *pal*-containing (Type IV FGI) loci. This suggests that, although the *Geobacillus* FGI pseudaminic acid biosynthetic proteins are more similar to each other than those encoded in the loci of *B. thuringiensis* ATCC35646 and *C. jejuni* 81–176, they may have distinct evolutionary origins and may have been derived through distinct horizontal gene transfer events (Additional file 2: Figure S2).

The Type V FGI strains *Geobacillus* sp. WSUCF1 and PSS1 encode orthologs of the glucose-1-phosphate thymidylyltransferase RmlA, thymidine diphosphate (dTDP)-glucose 4,6 dehydratase RmlB, dTDP-4-dehydrorhamnose 3,5-epimerase RmlC and dTDP-dehydrorhamnose reductases RmlD which together catalyse the sequential conversion of dTDP-D-glucose to dTDP-L-rhamnose [49], suggesting that the flagellin proteins in these strains are rhamnosylated. However, in *Geobacillus* sp. WSUCF1 the *rmlB* reading frame is disrupted by a transposon insertion (Fig. 1). The RmlABCD protein products of WSUCF1 (96.69% average amino acid identity) and PSS1 (79.24% average amino acid identity) share extensive sequence identity with the *rlmABCD* protein products responsible for glycosylation of the S-layer protein SgsE in *G. stearothermophilus* NRS2004/3a (AAR99610.1-613.1) [49]. This suggests genetic interchange between the glycan biosynthetic pathways for glycosylation of the two distinct surface components, the S-layer and flagellin proteins, may have occurred.

The FGI of *Geobacillus* sp. PSS1 also encodes orthologs of dTDP-6-deoxy-3,4-keto-hexulose isomerase (FdtA) and transaminase (FdtB). These enzymes catalyze the conversion of dTDP-6-deoxy-D-xylohex-4-ulose generated by RmlA and RmlB to dTDP-3-oxo-6-deoxy-D-galactose [50]. The *Geobacillus* sp. PSS1 proteins share 64.62% average amino acid identity with FdtA (AAS55720) and FdtB (AAS55722) in *Aneurinibacillus thermoaerophilus* L420-91^T^. In the latter strain, a third enzyme, FdtC, catalyzes the transfer of an acetyl group to dTDP-D-Fucp3N to form dTDP-D-Fuc3pNAc, which along with D-rhamnose forms the repeating unit of the S-layer glycan chain [50]. Orthologs of FdtB (44.41% amino acid identity to AAS55722), as well as the acetylase FdtC (AAS55722: 47.98% amino acid identity) are also found in the type II FGI strains *G. thermoglucosidans* Y4.1MC1 and TNO09.20. The absence of an ortholog of the isomerase FdtA suggests the FdtB and FdtC orthologs in these strains catalyse the transamination and acetylation of a distinct sugar, while the absence of FdtC orthologs in PSS1 suggests that the dTDP-3-oxo-6-deoxy-D-galactose of this strain is not acetylated.

Orthologs of the UDP-galactopyranose mutase (Glf), which catalyzes the conversion of UDP-galactose from its pyranose to its furanose form [51], are encoded in both Type III and five of the eight Type IV FGI strains. A partial *glf* gene is also encoded in the Type II FGI of *G. thermoglucosidans* DSM 2542^T^. Galactofuranose is found in the O-antigens of *E. coli* and *Klebsiella pneumoniae*, in the arabinogalactan main structural polymer in the *Mycobacterium tuberculosis* cell wall and the S-layer glycan of *Thermoanaerobacterium thermosaccharolyticum* [51, 52]. In the FGI region containing the *fdtC* and *fdtB* genes in the type II FGI strains *G. thermoglucosidans* TNO09.20 and Y4.1MC1, *G. thermoglucosidans* DSM 2542^T^ instead contains two genes, *tagD* and *pgs*, coding for a glycerol-3-phosphate cytidylyltransferase and a phosphatidylylglycerophosphate synthase (Pgs). The former enzyme converts sn-glycerol-3-phosphate to CDP-glycerol (E.C. 2.7.7.39), while Pgs catalyzes the conversion of CDP-diacylglycerol to phosphatidylglycerophosphate (E.C. 2.7.8.5) [53, 54]. The presence of these two key enzymes of phospholipid biosynthesis suggests that the flagellin in this strain may be modified with a phospholipid derivative. Lipid modification of surface proteins has only been identified in three haloarchaeal species to date; *Halifax volcanii, Halobacterium salinarum* and *Haloarcula japonica* [55]. Lipid modification of the flagellin in *G. thermoglucosidans* DSM 2542^T^ would, however, need to be confirmed experimentally.

### The *Geobacillus* FGIs show evidence of further glycan modifications

Aside from the distinct sugars observed in the glycans of the various flagellin-glycosylated bacterial taxa, the flagellin proteins and their glycan sugars are frequently heavily modified by formyl, methyl and acetyl groups [14]. While the biological functions of these modifications and the resultant structural diversity of the flagellin proteins and their glycans remain largely obscure, they may influence the functioning and roles of the flagellum [14]. Three distinct S-adenosylmethionine-dependent methyltransferases are encoded in the *Geobacillus* FGIs. The *sam1* gene in the Type I FGI of *G. thermoglucosidans* C56-YS93 is localised in the middle of the neuraminic acid biosynthetic locus, suggesting the encoded methyltransferase is responsible for methylation of this sugar. Five out of the eight type IV FGI strains contain a distinct methyltransferase (*sam2*), while *sam3* is located just upstream of the rhamnosyl biosynthetic genes of *Geobacillus* sp. WSUCF1 (Type V FGI). Methyltransferases of the FkbM family (*fkbM1*) are also present in the type III FGI strains *Geobacillus* sp. C56-T2 and PSS2, as well as the Type IV FGI strains *Geobacillus* sp. GHH01 and MAS1. A distinct FkbM-type methyltransferase (*fkbM2*) showing weak homology to *fkbM1* (30.80% average amino acid identity) is also encoded in the Type IV FGI strain *Geobacillus* sp. Et7/4. Methylated flagellin glycans have also been observed in the phytopathogen *Pseudomonas syringae* (rhamnosyl) and *Clostridium botulinum* (legionamic acid derivative) [19, 56]. The presence of two distinct families of methyltransferases in 15 of the 18 FGI^+^ strains suggests that flagellin and/or glycan methylation is an important feature of the flagella of *Geobacillus* spp. Formyltransferases are encoded in the flagellin glycosylation island of *Alteromonas macleodii* AltDE1 [57]. Similarly, three distinct formyltransferase genes are found in the three type I FGI strains (*fmt1*), both type III FGI strains (*fmt2*) and one type V FGI strain (*fmt3*). The *fmt3* gene in *Geobacillus* PSS1 occurs in the location occupied by the acetyltransferase gene *fdtC* in other dTDP-3-oxo-6-deoxy-D-galactose synthesising bacteria, suggesting that this sugar is formylated, rather than acetylated in *Geobacillus* sp. PSS1. The form and functions of the modifications derived by formylation and methylation of the flagellin proteins and/or the glycan chains in *Geobacillus* spp. remain to be structurally and functionally elucidated.

## Conclusions

Using comparative genomic approaches, we have identified and characterized the flagellin glycosylation islands in eigtheen *Geobacillus* strains for which genome sequences are available. These islands code for highly variable flagellin glycans comprising of several distinct sugar derivatives, which appear to be extensively diversified by the addition of methyl, acetyl and formyl groups. Extensive hallmarks of horizontal gene transfer, including divergent G + C contents and the presence of transposase and endonuclease genes, are present, suggesting that the versatility of these loci may be linked to their horizontal acquisition from distinct microbial origins.

The presence of FGIs in only half of the 36 compared *Geobacillus* strains raises questions on the functional roles of these glycans in members of this genus. Flagellin glycosylation is essential for flagellar filament formation and swimming motility in a range of Gram-negative bacterial taxa, as well as the Gram-positive relative *Paenibacillus alvei* [7, 8, 13]. The original descriptive publications of the genus *Geobacillus* indicated that the type strains of all the described species are all motile. This includes both *G. thermodenitrificans* DSM 465^T^ [20] and *G. caldoxylosilyticus* DSM 12041^T^ [58], which we have here shown lack FGI loci, suggesting that flagellin glycosylation is not a prerequisite for flagellum biogenesis and motility for members of this genus. This, however, assumes that the FGI^−^ and FGI^+^ *Geobacillus* strains compared differ only in terms of flagellin glycosylation. However, it should not be precluded that additional differences, such as differences in other flagellum biosynthetic genes, the differences in flagellin protein lengths and sequence homology among the FGI^−^ and FGI^+^ strains, may be contributing factors in filament biogenesis and flagellar motility.

In some Archaea, protein glycosylation is not essential for survival, but may make an adaptive contribution to survival in harsh environments [18]. Flagellin glycosylation was observed to increase the stability of flagellin proteins under heat treatment in the phytopathogen *P. syringae* pv. *tabaci*, while N-glycosylation of the *Bacillus amyloliquefaciens* (1,3-1,4)-beta glucanase was also shown to improve thermostability of this enzyme [17, 59]. The contribution of flagellin glycosylation to the thermostability of the flagellin protein in *Geobacillus* spp. is an attractive hypothesis. However, the temperature optima of both the FGI^+^ and FGI^−^ *Geobacillus* strains suggest a function for flagellin glycosylation other than thermostability. A large number of additional functions have been elucidated or hypothesised for flagellin glycosylation, including surface recognition, attachment, host defense avoidance and increased resistance against proteolytic cleavage [16]. Further analyses, such as knock-out mutagenesis and functional characterization of the flagellin protein and its glycan chain are needed to determine the function of flagellin glycosylation in members of the genus *Geobacillus*.

## Methods

### Characterisation of the flagellin glycosylation island loci

The flanking genes (*comFA* – BSU35470 and *raiA* - BSU35310) for the flagellar biosynthetic locus of *Bacillus subtilis* 168 (NC_000964.3) were used to identify the orthologous flagellum biosynthetic loci in the genomes of 36 *Geobacillus* isolates (Table 1). The loci were extracted from the genome sequences, open reading frames were predicted using GeneMark.hmm [60] and the G + C contents of the FGIs were determined using Bioedit v. 7.1.11 [61]. The proteins encoded on the FGIs were functionally annotated by BlastP comparison against the NCBI non-redundant (nr) protein database to identify orthologs in other bacterial taxa for which functional data is available. Orthology was assumed for those proteins sharing >50% amino acid identity over 70 % of the protein length. Further support for the protein function was obtained by identifying conserved functional domains through comparison of the proteins against the Conserved Domain Database using Batch CD-search [62]. Orthology among the proteins for the *Geobacillus* FGI datasets was determined using BlastP analyses in BioEdit [61] using the orthology criteria of >70% amino acid identity over 70% of the protein length.

### Phylogeny construction

Phylogenies were constructed on the basis of the *recN* house-keeping gene coding for the DNA repair protein RecN and the concatenated PseC and PseI amino acid sequences. Sequences were aligned using the MAFFT v. 7 alignment server [63] with default parameters. The *recN* Maximum Likelihood trees were constructed with the Molecular Evolutionary Genetics Analysis (MEGA) v. 7.0.14 software package [64], using the Tamura-Nei evolutionary model, complete gap deletion, nearest-neighbour-interchange ML heuristic method and bootstrap analysis (*n* = 1000). The concatenated PseC and PseI amino acid Maximum Likelihood phylogeny was likewise constructed with MEGA v 7.0.14 [64], using the Jones-Taylor-Thornton model, complete gap deletion, nearest-neighbour-interchange ML heuristic method and bootstrap analysis (*n* = 1000). A dendrogram was constructed on the basis of the presence/absence of orthologs for each of the FGI proteins among the FGI^+^ strains. Present orthologs were scored with a 1, while absent orthologs, as well as truncated and transposon-disrupted proteins were scored as a 0. The resultant matrix was used to generate a distance matrix using Bionumerics v 6.6 (Applied Maths N.V., Belgium) using absolute values and Pearson’s correlation. The distance matrix was used to generate an Unweighted Pair Group Method with Arithmetic Mean (UPGMA) dendrogram using Phylip v. 3.69 [65]. Similarity cut-off values of 50% were used to distinguish between the FGI types.

## Additional files

Additional file 1: Figure S1. Schematic diagram of the pseudaminic acid biosynthetic gene-containing FGIs. The pseudaminic acid biosynthetic genes are indicated in *green*. Flanking genes are indicated as *white* and *yellow* arrows. A scale bar indicates the predicted size of the regions. (PDF 23 kb)
Additional file 2: Figure S2. Maximum Likelihood phylogeny of the concatenated pseudaminic acid biosynthetic proteins PseC and PseI. Boot strap values (*n* = 1000 replicates) are indicated. (PDF 8 kb)

## Abbreviations

CDS: Protein coding sequence
FGI: Flagellin glycosylation island.
