# Supplementary figures and images for: Comparative genomic analysis of the flagellin glycosylation island of the Gram-positive thermophile Geobacillus
Source: BMC Genomics. 2016 Nov 14;17:913. doi: 10.1186/s12864-016-3273-2 (PMC5109656; doi:10.1186/s12864-016-3273-2)

5 kb

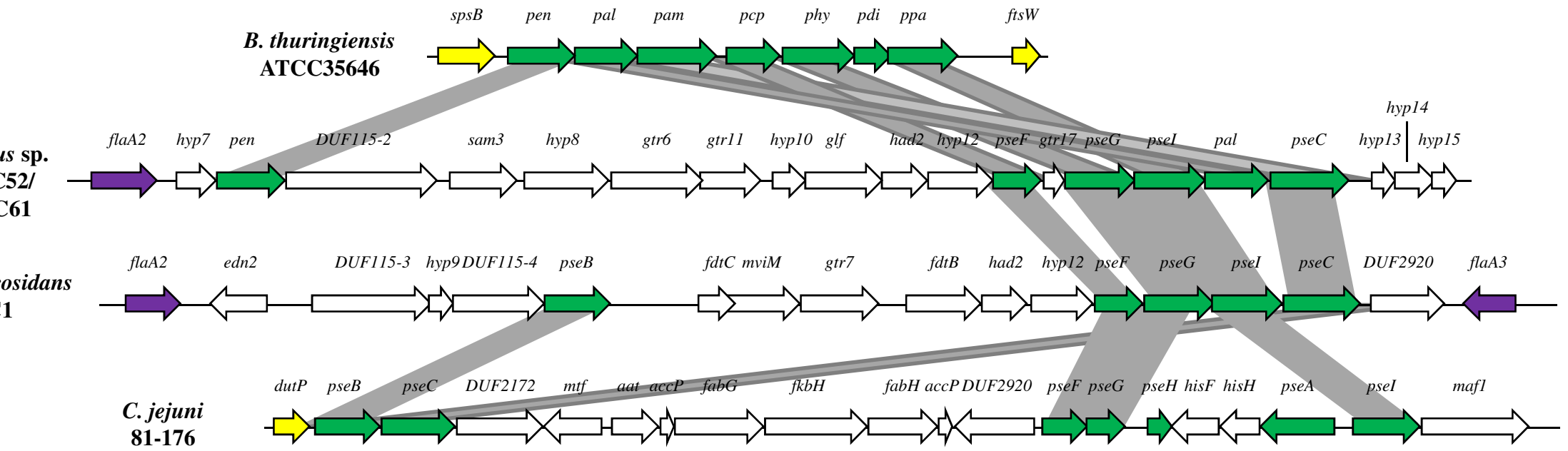

Supplement: Additional file 1: Figure S1. — Schematic diagram of the pseudaminic acid biosynthetic gene-containing FGIs. The pseudaminic acid biosynthetic genes are indicated in green. Flanking genes are indicated as white and yellow arrows. A scale bar indicates the predicted size of the regions. (PDF 23 kb) [file 12864_2016_3273_MOESM1_ESM.pdf]

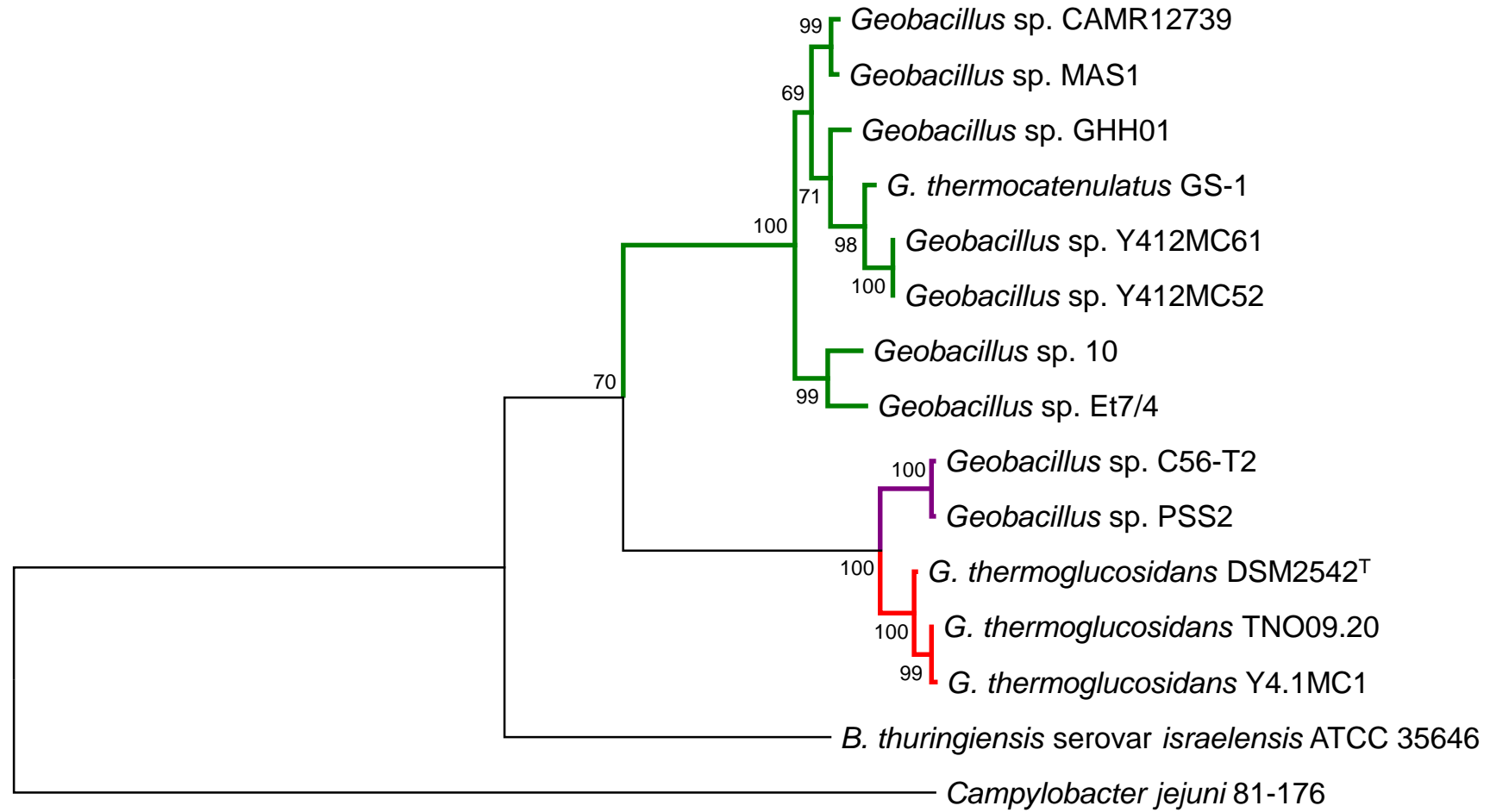

Supplement: Additional file 2: Figure S2. — Maximum Likelihood phylogeny of the concatenated pseudaminic acid biosynthetic proteins PseC and PseI. Boot strap values (n = 1000 replicates) are indicated. (PDF 8 kb) [file 12864_2016_3273_MOESM2_ESM.pdf]
